# Combining combing and secondary ion mass spectrometry to study DNA on chips using ^13^C and ^15^N labeling

**DOI:** 10.12688/f1000research.8361.1

**Published:** 2016-06-20

**Authors:** Armelle Cabin-Flaman, Anne-Francoise Monnier, Yannick Coffinier, Jean-Nicolas Audinot, David Gibouin, Tom Wirtz, Rabah Boukherroub, Henri-Noël Migeon, Aaron Bensimon, Laurent Jannière, Camille Ripoll, Victor Norris

**Affiliations:** 1 Equipe AMMIS, laboratoire MERCI EA 3829, faculté des Sciences et Techniques, University of Rouen, Mont-Saint-Aignan Cedex, France; 2 Institute of Electronics, Microelectronics and Nanotechnology (IEMN), UMR CNRS 8520, Lille1 University, Villeneuve d’Ascq, France; 3 Material Research & Technology Department (MRT), Luxembourg Institute of Science and Technology (LIST), Belvaux, Luxembourg; 4 Genomic Vision, Bagneux, France; 5 iSSB, Génopole, CNRS, UEVE, Université Paris-Saclay, Evry, France; 6 Laboratory Microbiology Signals and Environment EA4312, Department of Biology, University of Rouen, Mont-Saint-Aignan Cedex, France

**Keywords:** DNA isotope labeling, PCR, DNA extraction, silicon chip, surface coating, isotope recombination

## Abstract

Dynamic secondary ion mass spectrometry ( *D-SIMS*) imaging of combed DNA – the combing, imaging by SIMS or *CIS* method – has been developed previously using a standard NanoSIMS 50 to reveal, on the 50 nm scale, individual DNA fibers labeled with different, non-radioactive isotopes *in vivo* and to quantify these isotopes. This makes CIS especially suitable for determining the times, places and rates of DNA synthesis as well as the detection of the fine-scale re-arrangements of DNA and of molecules associated with combed DNA fibers. Here, we show how CIS may be extended to ^13^C-labeling via the detection and quantification of the ^13^C ^14^N ^-^ recombinant ion and the use of the ^13^C: ^12^C ratio, we discuss how CIS might permit three successive labels, and we suggest ideas that might be explored using CIS.

## Introduction

The CIS method (for combing-imaging by SIMS) combines DNA combing on a silicon surface, flooding the surface with cesium, and the sensitive imaging technique of dynamic secondary ion mass spectrometry (D-SIMS) as provided by a Cameca NanoSIMS 50, a machine that allows five or seven different masses to be detected simultaneously, depending on the version ^1, 2^. This method may be of interest for those who wish to obtain fine-scale, quantitative information on DNA replication and protein-DNA interactions at the level of single molecules without the need to modify these molecules. Previously, we have used the CIS method and a NanoSIMS 50 to obtain quantitative images of single DNA fibers combed on modified, SIMS-compatible, silicon surfaces with lengths similar to those expected for the B-conformation and with a resolution of 50 nm, i.e., 150 bp ^3^.

Variations in the rates and/or directions of DNA synthesis are implicated in many processes, including gene expression ^4^, nutrient sensing involving the alarmone (p)ppgpp ^5^, R-loop-mediated replication ^6^ and DNA repair ^7^. Variations between replisomes within the same chromosome have been observed using BrdU and light microscopy ^8^. To study such variations on the 50 nm scale, CIS might usefully be extended to image not only the ^12^C ^15^N ^-^ and ^13^C ^15^N ^-^ recombinant ions but also other ions. Here, we describe in detail the protocols needed for CIS, we report using CIS to image the ^13^C ^-^, we suggest ways in which ^13^C-imaging could be improved, and we mention possible applications.

## Materials and methods

### Preparation of DNA

In principle, DNA could be extracted from any organism to perform CIS. DNA generated *in vitro* can also be used. For the experiment reported here, for example, we extracted DNA from the 168 ( *trpC2*) strain of *Bacillus subtilis* and a derivative (MT119, *trpC2 leuB6 r- m-*) harboring a 21.1 kb long, chloramphenicol-resistant plasmid (pHV1431 plus insert of 10.3 kb) ^3^. On modified silicon surfaces at concentrations of 0.2 µg/mL, most of the DNA is combed as single fibers. Higher concentrations can lead to fibers running together whilst lower concentrations result in an insufficient number of fibers on the images. Combing works well with buffers containing NaCl at ionic strengths of around 0.2M.

Bacterial culture medium: 14g/L of K _2_HPO _4_, 6g/L of KH _2_PO _4_, 1g/L of sodium citrate, 10mM MgSO _4_, 0.01% (w/v) of tryptophan, 0.005% (w/v) of leucine, 0.2%(w/v) of ^13^C-glucose (Isotec) and 0.8g/L of ^15^NH _4_Cl (Isotec). Lysis buffer: 50mM of Tris-HCl (pH8.0), 10mM of EDTA (pH8.0), 150mM of NaCl and 5mg/ml of lysozyme. Proteinase K: Final concentration of 0.2mg/mL (Roche). Sarcosyl Buffer: Final concentration of 1.2%. Phenol/chloroform: Solution PCI: phenol-chloroform-isoamylalcohol (25:24:1 v/v) (the phenol is first saturated in NaCl 150mM and buffered at pH of about 7); Solution CI: chloroform-isoamylalcohol (24:1 v/v). RNase: Final concentration of 20µg/mL (Roche).

Other materials required are: 100% cold ethanol; 70% (v/v) ethanol; NaCl solution: 0.2M to 1M; PureYield ^TM^ plasmid midiprep system (Promega); restriction enzyme; 0.3M Potassium acetate pH7.0; TaKaRa EX Taq ^TM^ system (TaKaRa); Expand Long Template PCR System (Roche Applied Science); QIAquick PCR Purification kit (QIAGEN).

***DNA preparation from bacterial cultures.*** DNA can be prepared from any cells using a procedure that removes contaminating peptide and RNA molecules. In the case of the labeling and extraction of *B. subtilis* DNA, *B. subtilis* cells were cultivated at 30–37°C for ~20 generations in bacterial culture medium containing stable isotopes (here, ^13^C and ^15^N, see above) to saturation. Then, to prepare chromosomal DNA free of peptides and RNA, one milliliter of a freshly saturated culture was centrifuged at 15000 g for 2 min at 4°C. The supernatant was discarded. Pelleted cells were resuspended in 0.5 mL of lysis buffer. The cell resuspension was incubated for 20 minutes at 37°C. Proteinase K was added to a final concentration of 0.2 mg/mL in addition to 20µL of sarcosyl buffer 30% (1.2%, final concentration) and incubated for 20 min at 65°C. To remove peptides and cell fragments by a phenol/chloroform treatment, we lowered the temperature of the sample on ice for 3 min and then added 500µL of solution PCI (see **Preparation of DNA**) at 4°C and vortexed strongly for 30 s. The mixture was centrifuged at 15000 g for 15 min at room temperature in a benchtop centrifuge. The aqueous solution was recovered and 500 µL of solution CI was added to it (see **Preparation of DNA**). This mixture was vortexed strongly and centrifuged as above. RNase was added to a final concentration of 20 µg/mL to the aqueous, nucleic acid-containing phase and incubated for 10 min at 37°C. DNA was purified by a second phenol/chloroform extraction as above. We added 2.2 volume of 100% -20°C ethanol to the aqueous solution and centrifuged at 15000 g for 20 min at 4°C in a benchtop centrifuge. The supernatant was discarded and the DNA pellet was washed in 70% cold ethanol. This mixture was centrifuged again for 10 min at 4°C at 15000 g and the supernatant was discarded. The DNA pellet was dried for 10 min under vacuum and resuspended carefully in pure water for at least 12 h at room temperature (note that incomplete solubilization of ethanol-precipitated DNA severely perturbs combing). DNA concentration was measured by absorbance at 260/280 nm using a Nanodrop 2000 (Thermo Scientific). Depending on the fragment length, DNA concentration should be adjusted to within the range 0.2 to 2 µg/mL by dilution in NaCl solutions that may range up to to 1M; here, we used DNA at 0.2µg/mL in 0.2M NaCl.

In this paper, we only report CIS in the case of chromosomal DNA. CIS can also be used to analyse plasmid DNA (not shown). In the case, for example, of a 20 kb plasmid from *B. subtilis*, plasmid DNA may be extracted using the PureYieldTM plasmid midiprep system (Promega Corporation, Madison, WI, USA), followed by linearization of the plasmid by a single cutter restriction enzyme according to suppliers’ instructions (e.g. New England BioLabs, Inc., Hitchin, UK) to give fragments of the appropriate size; this linearization is needed before combing because of the difficulty of combing supercoiled circular molecules. After linearization, the DNA can be purified by the phenol/chloroform procedure and recovered by ethanol precipitation in the presence of 0.3 M potassium acetate pH 7.

***DNA preparation by PCR (protocol used to prepare PCR-generated DNA fragments 1-20 kb long).*** DNA fragments generated by PCR can be analyzed by CIS ^3^. We routinely produce fragments <5 kb using the TaKaRa Ex Taq ^TM^ system (Takara Shuzo Co., Ltd, Shiga, Japan). For products of 5-20 kb, we use the Expand Long Template PCR System from Roche Applied Science (Mannheim, Germany). The PCR products can be purified with the QIAquick PCR Purification kit (QIAGEN GmbH, Hilden, Germany). PCR products can then be diluted for CIS as described above.

### Preparation of silicon surfaces

This experiment required an efficient fume hood. The materials used were: P-type 100 crystalline silicon wafers (Siltronix) with a resistivity > 1 Ω cm (typically 10 mm × 10 mm × 380 μm); Piranha solution obtained by mixing 3 volumes of 96% of sulfuric acid [VLSI (very large-scale integration)-grade] and 1 volume of 30% hydrogen peroxide (VLSI-grade), to be used immediately; 50% hydrofluoric acid (VLSI-grade); acetone; isopropyl alcohol ( *i*PrOH); chloroform (CHCl _3_); ethanol (EtOH); 1-tetradecene; a photochemical reactor for UV irradiation (λ=312 nm) (our home-made reactor has 8 tubes in a circle around a Schlenk tube with a fan at the bottom to limit any increase in temperature); a goniometer system (DIGIDROP, GBX, France).

***Preparation of hydrogenated-terminated silicon surfaces.*** In the following preparation, it should be noted that the piranha solution is a powerful oxidant that reacts violently with organic materials; it can cause serious skin burns and must be handled with great care in a well-ventilated fume hood while wearing appropriate chemical safety protection. Moreover, HF is a hazardous acid that can cause severe damage to tissues if burns are not treated properly. Etching of silicon should therefore be carried out in a fume hood with the right safety measures, which include a face shield and double-layered nitrile gloves.

The silicon surface was cleaned in an ultrasonic bath for 5 min periods in acetone and isopropylic alcohol. It was then rinsed extensively with ultrapure water and immersed for 20 min in a piranha solution to remove organic contaminants on the surface. This was followed by the immersion of the clean surface in an aqueous solution of HF (50%, as provided by the supplier) to generate a hydrogen-terminated surface (Si-H) ^9^. This surface was washed extensively with water and then dried by blowing with nitrogen. The resulting surface typically had a contact angle of 85° for a 1 µL water droplet. Note that freshly prepared Si-H surfaces were used immediately for DNA combing or for grafting hydrocarbon chains (see below). Before combing, coated silicon surfaces were protected from dust and dried because a thin film of water greatly reduces DNA adsorption.

***Formation of organic monolayers on hydrogen-terminated silicon surfaces.*** To obtain highly hydrophobic surfaces, alkenes with C _14_ alkyl chains were grafted onto freshly prepared Si-H surfaces using the hydrosilylation reaction of 1-tetradecene with the hydrogen-terminated silicon surface ^9^. The freshly hydrogen-terminated silicon surface (see above) was immersed in a Schlenk tube containing 10 ml of previously deoxygenated, neat 1-tetradecene under nitrogen bubbling. This was then irradiated at 312 nm in a photochemical reactor for 3 h. We removed unreacted and physisorbed 1-tetradecene by rinsing with chloroform and ethanol at room temperature. The silicon substrate was dried under a stream of nitrogen. We verified that water contact angles (using deionized water in the ambient atmosphere at room temperature) are around 104° as measured to an accuracy of ± 2° with a remote, computer-controlled, goniometer system (N.B., protect the surface from dust, see above).

***Combing on silicon.*** The 'drop' and 'lift' methods ^10, 11^ can both be used to comb DNA on the Si-C _14_ surface. Both 'drop' and 'lift' methods entail the DNA becoming attached to the surface and then drawn out and aligned perpendicular to the triple line or the meniscus, respectively. The ‘drop’ method entails depositing 10 µL of prepared DNA solution on the silicon surface, incubating it for 10 min on the bench, tilting the surface with tweezers to 45° to cause the drop to roll off the surface, and washing the surface by immersion in water and rapidly air-drying; the quality of combing via this method can be adversely affected by vibrations occurring during the movement of the drop. Here, we used the ‘lift’ method, which entails immersing a part of the silicon surface in a DNA solution (see above); after 5 min incubation, the silicon surface was pulled out of the solution at a constant speed (600 µm/min) and rapidly air-dried. In both ‘drop’ and ‘lift’ methods, washing the surfaces after combing helps avoid perturbation of D-SIMS analysis by saline crystals. The tightly controlled, motor-driven ‘lift’ method gives more reproducible, better quality results but takes longer and requires more DNA than the manual ‘drop’ method. The ‘lift’ method is better than the drop method for fragments less than 5 kb; the ‘drop’ method is satisfactory with fragments equal to or greater than 5 kb.

### D-SIMS methods

***Cs flooding method.*** To avoid a premature destruction of thin samples before SIMS detection ^12^, a cesium flooding system is recommended to deposit neutral cesium on samples ^13, 14^. We used a UHV Cs evaporation system which is available for purchase from the Luxembourg Institute of Science and Technology (LIST) . It comprises a neutral cesium evaporator and an independent stand-alone UHV chamber. A suitcase under UHV (10 ^-8^ to 10 ^-10^ Torr) was used to transfer samples to the NanoSIMS50 thereby avoiding an immediate reaction between the neutral cesium deposit and air. To analyze combed DNA (as performed here), we flooded the surface with Cs ^0^ at 1.5 Å/s for 1800 s before SIMS imaging. An alternative to cesium flooding is to coat samples with Au; a Cressington Sputter Coater can be used to deposit a layer of Au, approximately 60 nm thick, on wafers with combed DNA but the quality of the images subsequently obtained is often poor.

***NanoSIMS 50 analyses.*** In SIMS analyses of samples labeled with carbon and nitrogen, there are several factors that should be borne in mind. First, carbon can be detected as C ^-^ and in multi-clusters that include CN ^-^ and C _2_^-^ (N.B. CN is easier to ionize and therefore detect than C and C _2_ because the electron affinity is 3.8eV for CN ^-^, 1.26eV for C ^-^ and 3.27eV for C _2_^-^). Second, nitrogen can be detected in the multi-cluster CN (note that nitrogen forms fewer types of multi-clusters thant carbon even though it can form to a very limited degree multi-clusters that include NO, N _2_H, and NS). Third, the probability of formation of the CN multi-cluster in the mixing-recombination under the primary beam is related to the distance between the carbon and nitrogen atoms ^15^; this means that differentially labeled macromolecules can be colocalized if they are within 2 nm of one another; it also means that the carbon and nitrogen in the DNA are more likely to recombine with one another than with carbon and nitrogen that are further away. Note that even a trace amount of contaminants is an important problem for the highly sensitive CIS method and strenuous efforts should therefore be taken to protect samples from contamination by, for example, an atmosphere containing C and N. Silicon surfaces with combed DNA were analyzed in the multi-collection image mode of the NanoSIMS 50 (Cameca, Gennevilliers, France). The NanoSIMS 50 was used in the negative secondary-ion mode with the Cs ^+^ primary ion beam. In this case high spatial resolution images were obtained using a primary beam around 0.5–1.0 pA in intensity and an impact energy of 16 keV. The surface was rastered by the cesium beam on a surface from 5 × 5 to 15 × 15 µm ^2^. The instrument was tuned to limit the dispersion in aperture and energy in order to obtain a minimal mass resolution of M/ΔM = 5000 (10% height peak measurement).

Aphelion 3.2 was used to analyze the results pixel by pixel or line by line, whilst ImageJ plus the OPEN MIMS plug-in (which opens the .im format ^16^ and is available from http://www.nrims.harvard.edu) was used to analyze the data in an ROI (Region Of Interest). The data were summed and the images colored using WinImage 2. The images of the ^12^C (m = 12.000000), and/or ^13^C (m = 13.00335484), ^12^C ^14^N (m = 26.003074), ^12^C ^15^N (m = 27.00010898) or ^13^C ^14^N (m = 27.00642885), ^13^C ^15^N (m = 28.00346382) were acquired simultaneously in 256 × 256 pixels with a dwell time of 10 ms per pixel. Note that ^12^C ^15^N and ^13^C ^14^N cannot be detected *simultaneously* in the same ‘sputter section’. The sulfur ^32^S (m = 31.9720718) distribution can be also acquired to control the quality of the preparation.

## Results

Raw data for Combing-Imaging by SIMS of bacterial DNA Double-labeled (13C, 15N) chromosomal DNA from *Bacillus subtilis* was combed onto a silicon surface, analysed with a NanoSIMS 50 set to detect masses of 12, 13, 26, 27 and 28 for 12C-, 13C-, 12C14N-, 13C14N- and 13C15N- ions, respectively. The data were treated with Aphelion Click here for additional data file. Copyright: © 2016 Cabin-Flaman A et al. 2016 Data associated with the article are available under the terms of the Creative Commons Zero "No rights reserved" data waiver (CC0 1.0 Public domain dedication).

**Figure 1.**
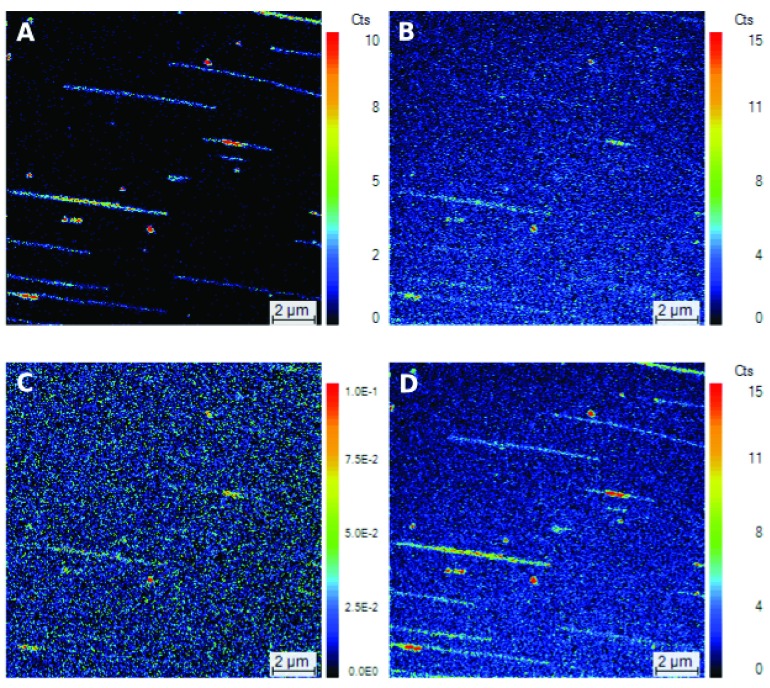
SIMS images of double-labeled ( ^13^C, ^15^N) chromosomal *Bacillus subtilis* DNA. The DNA was combed at 0.2 μg/mL on a Si_C14 wafer using the lift method, the wafer surface was covered with cesium and analyzed with a NanoSIMS 50. Primary beam intensity, 1 pA; dwell time, 30 ms; field of view, 15 μm × 15 μm; 256 × 256 pixels; scale bar = 1 μm. ( **a**) ^13^C ^15^N, ( **b**) ^13^C ^14^N, ( **c**) Isotope ratio ^13^C/ ^12^C, and ( **d**) ^13^C ^15^N+ ^13^C ^14^N. ( **a**) and ( **b**) are the results of adding the counts from three successive sputter sections, ( **c**) is the ratio between the two sets ( ^13^C and ^12^C) of three successive sputter sections, and ( **d**) is the three sputter sections of ( **a**) plus the three of ( **b**). The count numbers of ( **a**), ( **b**) and ( **d**), and the count ratio of ( **c**) are given on the linear color scales.

The CIS technique reveals individual DNA fibers of a wide variety of lengths lying in parallel ( Figure 1). These fibers are particularly clear in the ^13^C ^15^N image of the DNA ( Figure 1a) but, importantly, they can also be discerned in the ^13^C ^14^N image ( Figure 1b). The images differ because (1) the coating on the silicon surface contains a large amount of the natural isotopes of carbon of which 1% is ^13^C; this 1% can then recombine with contaminant nitrogen to generate a high background of ^13^C ^14^N that partially masks the signal from the labeled DNA (even though this DNA is 99% ^13^C) and (2) the background of ^13^C ^15^N is low since this ion arises at a frequency of 1:25000 in combinations of naturally occurring carbon and nitrogen).

Most of the fibers have similar counts throughout their lengths but, in a couple of cases, two fibers overlap and this results in more ^13^C ^15^N ^-^ recombinant ions being detected (the red stretches in Figure 1a). The relative constancy of the counts and the similarity between the calculated lengths of the DNA and the lengths estimated by SIMS support the claim that CIS does not stretch or condense DNA ^3^. However, an exception to this may occur when the DNA is so short that it cannot be combed properly, as is probably the case of the red spots of a few nm ( Figure 1a).

The fibers in the image of the isotope ratio ^13^C/ ^12^C image ( Figure 1c) are difficult to detect in their entirety. One reason for this difficulty is that the natural carbon in the coating on the surface contains ^13^C, as mentioned above. A second reason is that the yield of carbon in the C ^-^ form is relatively low and therefore the ratio can fluctuate (see Materials and methods). A third reason is that the carbon in the DNA reacts with neighboring atoms in the DNA (and elsewhere) such as nitrogen – and indeed carbon itself – to give multi-clusters (see Materials and methods); this means that much of the ^13^C is being distributed into multi-clusters that include ^13^C ^15^N, ^13^C ^14^N, ^12^C ^13^C and ^13^C ^13^C. It is therefore significant that, even in these unfavorable conditions for detecting ^13^C via the ^13^C: ^12^C ratio, ^13^C-labeled fibers can just about be distinguished ( Figure 1c).

In the experiment reported here, 98% of the nitrogen in the DNA is ^15^N. This means that the counts of ^13^C ^14^N coming from recombination between the atoms within the DNA are nearly 98% lower than they would be if all the nitrogen in the DNA were present as the ^14^N isotope. To answer the question of whether labeling with ^13^C alone (i.e., without ^15^N-labeling) is sufficient to allow DNA to be detected, we added the counts of ^13^C ^15^N ( Figure 1a) to those of ^13^C ^14^N ( Figure 1b) to obtain an image of ^13^CN ( Figure 1d). The fibers are again clear, showing that ^13^C labeling is indeed sufficient to allow DNA to be detected.

## Discussion

Previously, we have suggested that CIS could be used in conjunction with pulse-labeling with different isotopes to identify origins of replication or to study local variations in the rate of DNA elongation resulting from signals generated inside or outside cells or from addition of drugs ^3^. Such pulse-labeling can be readily based on ^15^N since this is relatively rare naturally (0.36%) and enriching in ^15^N therefore makes fibers readily detectable. Here, we ask whether ^13^C labeling with ^13^C alone might suffice for detection via CIS. We try to answer this question in the context of having labeled the fibers with both ^13^C and ^15^N ( Figure 1a). Even in these conditions, the image of the ^13^C ^14^N ^-^ recombinant ion does allow fibers to be discerned, albeit with difficulty ( Figure 1b). This difficulty is due not only to reduction of the ratio by surface contamination by carbon (which contains 1% ^13^C) but also to the recombination that generates multi-clusters (see Materials and methods); the latter includes recombination between the ^13^C and ^15^N in the fibers to give ^13^C ^15^N (it is therefore not surprising that the fibers are hard to detect in Figure 1c using the ratio of the ^13^C ^-^ and ^12^C ^-^ ions). Another way to decide whether ^13^C ^14^N can be used for imaging when DNA is labeled with just ^13^C is to add the counts from both ^13^C ^14^N and ^13^C ^15^N by combining the results in Figure 1a & 1b so as to increase the counts of ^13^C ( Figure 1d). This shows that the fibers can indeed be discerned (although it should be noted that here both strands are labeled rather than just one strand being labeled as would occur after a short pulse). This result is significant because it means that, in the same experiment, one could label the DNA successively: first with ^13^C in ^14^N medium to detect ^13^C ^14^N and then with ^15^N addition to detect ^13^C ^15^N. One might even succeed in detecting three consecutive sequences, for example, by (1) labeling with ^13^C (via U- ^13^C-Glc) to detect the ^12^C ^13^C ^-^ or ^13^C ^13^C ^-^ recombinant ion (or indeed simply the ^12^C ^-^ and ^13^C ^-^ ions), then by (2) labeling with ^15^N (via ^15^NH _4_Cl) to detect ^13^C ^15^N, and finally by (3) replacing either the ^13^C with ^12^C (via U- ^12^C-Glc) to detect ^12^C ^15^N or the ^15^N with ^14^N (via ^14^NH _4_Cl) to detect ^13^C ^14^N. Such consecutive labeling would allow investigation of local variations in the velocities of replication.

In principle, CIS could permit testing of several hypotheses. These include (1) the idea that the elongation step of DNA replication is coupled to central carbon metabolism ^17– 19^ and that this changes the local structure of the DNA ^20, 21^ or changes the relative copy numbers of genes ^4^ and thereby affects the phenotype, (2) the idea that the strand separation resulting from ion decondensation is responsible for the initiation of DNA replication ^22^, (3) the idea that the time to rereplicate the chromosomes in a cell population growing in steady state is highly variable since these populations are phenotypically diverse ^23, 24^, (4) the idea that the replication of a particular species of bacteriophages or viruses is very diverse. CIS could also be used to study the fine-scale interaction between DNA sequences and RNA, proteins and polyamines. Finally, CIS might eventually be used to study the modification of individual DNA fibers due to the covalent addition of methyl and acetyl groups, sugars, and other molecules, always providing that these molecules could either be labeled specifically by very rare isotopes such as ^14^C or bound specifically by antibodies or aptamers that were themselves labeled.

The value of such potential applications of CIS depends on the number of different ions that can be detected and on the quality of this detection (which depends on factors such as the signal:background ratios, the dwell time, the intensity of the primary beam, and the use of serial sputter sections). With the latest NanoSIMS 50L version of the NanoSIMS range, seven different masses can be detected simultaneously in the acquisition of a single ‘sputter scan’ ^25^, which increases the numbers of carbon multi-clusters that can be analyzed. Finally, the quality of detection of ^13^C ^-^ and the recombinant ^13^CN ^-^ ions could be improved by coating the silicon surface with carbon depleted in ^13^C. In principle, therefore, CIS could distinguish between DNA labeled so as to give consecutive stretches enriched in (1) ^13^C (detectable as ^13^C ^13^C), then (2) ^13^C and ^15^N (detectable as ^13^C ^15^N) and, finally, (3) ^12^C (detectable as ^12^C ^15^N) (note there are other possible combinations). CIS could, of course, be extended further still if the DNA were labeled with Br or I or even ^14^C ^26^.

## Conclusion

CIS combines the advantages of DNA combing and SIMS in providing high sensitivity and high resolution since DNA fragments down to 1500 nm can be imaged at a resolution of 50 nm. Moreover, the labeling with stable isotopes does not significantly perturb cells. The fact that CIS can detect ^13^C ^14^N increases the number of separate labels available to the technique, thereby making CIS particularly valuable for studying phenotypically important variations in the elongation of DNA replication, as well as the processes of initiation of replication, recombination and repair. The increase in the repertoire of labels available to CIS also supports the case for it being adapted to study the interaction between DNA and other molecules, including proteins and drugs, and perhaps even to the precise localization of covalent modifications to DNA.

## Data availability

The data referenced by this article are under copyright with the following copyright statement: Copyright: © 2016 Cabin-Flaman A et al.

Data associated with the article are available under the terms of the Creative Commons Zero "No rights reserved" data waiver (CC0 1.0 Public domain dedication). 

***F1000Research*: Dataset 1.** Raw data for Combing-Imaging by SIMS of bacterial DNA, 10.5256/f1000research.8361.d120278 ^27^
