# Supplementary material for: Combining combing and secondary ion mass spectrometry to study DNA on chips using 13C and 15N labeling
Source: F1000Res. 2016 Jun 20;5:1437. [Version 1] doi: 10.12688/f1000research.8361.1 (PMC4943295; doi:10.12688/f1000research.8361.1)
Supplement: Peer review history

# Peer review: Referee response for version 1

**DOI:** 10.5256/f1000research.8991.r14893

**Published:** 2016-07-11

**Authors:** Freddy C. Adams

**Affiliations:** 1 Department of Chemistry, University of Antwerp, Antwerp, Belgium

The article is a well-written report on work concerning the use of the Cameca NanoSims50 instrument for imaging of DNA fibers immobilized on silicon substrates. The work is a follow-up of earlier papers on the same subject by the present consortium. It contains work that is original and of interest for the scientific community. It shows the value of potential applications of Combing-Imaging with SIMS (CIS) and leads the way to further work with this ultra-sensitive chemical imaging tool. I fully support its indexations.

I have read this submission. I believe that I have an appropriate level of expertise to confirm that it is of an acceptable scientific standard.

# Peer review: Referee response for version 1

**DOI:** 10.5256/f1000research.8991.r14618

**Published:** 2016-06-27

**Authors:** Bruno Fabre

**Affiliations:** 1 MaCSE Research Group, Institute Of Chemical Sciences Of Rennes, UMR CNRS 6226, University of Rennes 1, Rennes, France

This article provides significant insights in a novel SIMS imaging technique focusing on the imaging of DNA fibers immobilized on modified silicon surfaces. The conclusions are adequately supported by the experimental data. Consequently, I recommend indexation of this as is.

I have read this submission. I believe that I have an appropriate level of expertise to confirm that it is of an acceptable scientific standard.
